# Design of a mixed reality–based digital twin human–machine interaction system for special working conditions

**DOI:** 10.3389/frobt.2026.1774317

**Published:** 2026-04-15

**Authors:** Shuai Li, Yifei Hu, Huiyu Li, Junjie Xu, Ming Li, Yunqiang Zhang, Qingting Ren

**Affiliations:** 1 State Grid Ruijia (Tianjin) Intelligent Robot Co., Ltd., Tianjin, China; 2 School of Mechanical Engineering, Zhejiang University, Hangzhou, China; 3 State Grid Tianjin Electric Power Company, Tianjin, China

**Keywords:** digital twin, human-computer interaction, human-machine collaboration, mixed reality, special working conditions

## Abstract

Operational robots have demonstrated significant potential in complex scenarios such as live-line maintenance and medical surgery. Existing research on Mixed Reality (MR) and Digital Twin (DT) systems has primarily focused on unidirectional data visualization and passive state monitoring. Existing research on Mixed Reality (MR) and Digital Twin (DT) systems has primarily focused on unidirectional data visualization and passive state monitoring, acting as “open-loop” observation tools that fail to address low operational precision and inefficient human-robot synergy in dynamic, high-risk environments. For the first time, we integrate an MR-based closed-loop digital twin operating system for human-robot collaborative operation into the task execution of live-line operation equipment to address the above challenges. Moving beyond simple visualization, the proposed framework establishes an integrated operational paradigm that bridges the gap between immersive perception and real-time interventional control. This framework comprises three integral components: (1) the construction of a high-fidelity virtual digital twin; (2) the development of a human-computer interaction paradigm based on MR technology; and (3) the establishment of an MR-based human-machine collaborative operation mode. Building upon this framework, a system was implemented for live-line working robots. Experimental results indicate that, compared with traditional control methods, the proposed system reduces the task completion time of live-line equipment tasks by 14.3% on average, verifying the feasibility and effectiveness of the pioneering application of the closed-loop digital twin operating system in live-line operation equipment.

## 1 Introduction

With the rapid advancements in technologies such as mixed reality, information communication, and natural human-computer interaction, the demand for enhanced intelligence and autonomy in industrial robots is attracting intense attention. Conventional industrial robot systems lack the adaptability to operate in dynamic environments and cannot complete tasks via autonomous learning. The traditional method of programming an industrial robot, which involves manually designing a path for a specific task, is a time-consuming, laborious, complex, and error-prone process. Furthermore, this approach necessitates a deterministic environment where all factors are fully defined and precisely positioned. Consequently, if any aspect of the design or the operational environment is altered, the robot must be entirely reprogrammed (Arents and Greitans, 2022). Thus, for robots to execute complex operational tasks in environments beyond controlled settings such as factory floors, human teleoperation is typically required. Operating under conditions of uncertainty remains one of the central challenges confronting conventional robotics (Kemp et al., 2007). In complex equipment operation scenarios such as live-line maintenance, mine clearance and explosive ordnance disposal, and surgical procedures, this issue becomes particularly evident. In recent years, highly intelligent and autonomous robotic systems designed for special working conditions have played an increasingly important role in performing high-risk tasks (Wong et al., 2017). However, most of these operational systems currently provide visual feedback through two-dimensional (2D) displays. In such operation modes, a large number of user errors and human–machine interaction issues related to the equipment itself have been observed (Barua and Sau, 2022). To address this issue, it is necessary to introduce new technologies and interaction paradigms that can ensure accurate visual feedback and information processing within operational systems, thereby enhancing task efficiency and overall safety.

Digital twin technology enables precise mapping and prediction of physical entities through real-time data integration and high-fidelity modeling, thereby optimizing task allocation, improving production efficiency, and ensuring operational safety. As research in this field continues to advance, digital twin technology—first proposed by Professor Michael Grieves in the United States (Grieves and Vickers, 2016)—has begun to emerge as a transformative approach across a wide range of engineering domains. Rochdi Kerkeni and colleagues describe a data-based DT architecture for the monitoring, and predictive maintenance (PdM) in manufacturing (Kerkeni et al., 2024). In recent years, as a comprehensive embodiment of next-generation information technologies, digital twin technology has achieved remarkable results in various fields such as industrial manufacturing (Tao et al., 2018) and aerospace engineering (Stevens, 2023). For example, Arne Bilberg and colleagues utilized digital twin technology to allocate tasks according to the respective skills of humans and robots, plan optimal motion trajectories for robots, dynamically balance workloads between the two during assembly processes, and automatically generate robot programs (Bilberg and Malik, 2019). Although these studies have verified the application value of DT in industrial manufacturing and assembly, their DT architectures only focus on passive monitoring or task planning, and no closed-loop operating system with real-time command issuance, physical execution, and state feedback has been designed. Moreover, all existing DT/MR systems are not adapted to the high-risk characteristics of live-line operation equipment, leaving a blank in the application of closed-loop digital twin technology in this field.

Mixed reality (MR) technology represents an interactive paradigm in which vision serves as one of the most critical input channels. Over the years, research on human-computer interaction in mixed reality environments has been continuously seeking more natural and efficient interaction methods (Azofeifa, 2022), aiming to mitigate the limitations of traditional systems through stereoscopic and multidimensional scene-based inputs (Li et al., 2022). By leveraging mixed reality technology, users can obtain real-time operational information in transparent and visualized multimodal environments, thereby compensating for the cognitive shortcomings of conventional operation modes and enabling more intuitive and natural human–machine interaction. At present, mixed reality technology has begun to find applications in specialized scenarios such as remote medical surgery (Coelho, 2022), extravehicular operations in space (Torrence and Dressel., 2025), and mine clearance and explosive ordnance disposal (Canaza Cca et al., 2024). A research team from South China University of Technology employed a Leap Motion sensor to capture the operator’s hand movements (Gallala et al., 2022) and convert them into robotic control commands. Through mixed reality technology, the system provides real-time visual feedback of the robot’s working environment, thereby ensuring operational safety and efficiency while enhancing the overall human–machine interaction experience (Luu et al., 2025).

The above research progress indicates that digital twin and mixed reality technologies hold significant application potential in the field of human–robot interaction under special working conditions. However, existing studies still suffer from two critical limitations: firstly, most MR-DT integrations only serve as open-loop visualization or monitoring tools, and no systematic closed-loop digital twin operating system has been developed for real-time interventional control; secondly, none of the existing MR-DT studies have been applied to the task execution of live-line operation equipment, a typical high-risk special working condition with strict requirements for real-time feedback and closed-loop control. These limitations result in insufficient improvement in task efficiency and operational safety for high-risk industrial equipment. Therefore, this study for the first time develops a mixed reality-based closed-loop digital twin operating system and applies it to the task execution of live-line operation equipment.

## 2 Design of a digital twin system based on mixed reality

### 2.1 Analysis of human–computer interaction requirements in mixed reality

In complex equipment operation scenarios under special working conditions such as live-line maintenance, explosive ordnance disposal, and surgical procedures, traditional equipment control modes have long faced critical challenges characterized by low operational precision, high safety risks, and poor collaborative efficiency. These issues have become key bottlenecks hindering the improvement of operational quality and safety management. On the one hand, the operation of real industrial equipment—such as live-line maintenance robots and heavy robotic manipulators—still heavily relies on human experience. Operators must observe the equipment status on-site and manually adjust their actions, which easily leads to operational deviations caused by visual blind spots or reaction delays. For instance, during high-voltage wiring tasks, when operators judge the alignment between a robotic arm and a conductor solely by visual observation, errors such as unintended collisions or loose connections may occur. These not only compromise task quality but also pose significant risks of equipment damage and safety accidents. On the other hand, traditional control modes lack real-time data feedback and intelligent intervention mechanisms, making it difficult to promptly detect abnormalities in equipment operating parameters. Moreover, single-person operation cannot effectively manage multi-device coordination, resulting in low overall workflow efficiency. These limitations become even more pronounced in complex working environments such as outdoor or high-altitude operations, where the constraints of manual control are further amplified.

With the advancement of industrial digitization and intelligent control technologies, the construction of a human–machine interaction system capable of achieving precise command execution and real-time closed-loop feedback has become an inevitable choice for addressing the aforementioned challenges. Relevant studies have demonstrated that, compared with on-site operations under traditional hazardous working conditions, mixed reality teleoperation based on a digital twin system can eliminate potential safety hazards (Perez et al., 2025) and enhance human-machine collaboration efficiency (Subramanian et al., 2024). By enabling standardized command generation and protocol conversion, the operator’s control intentions can be accurately translated into machine-readable instructions, thereby minimizing subjective errors inherent in manual operation. Supported by real-time communication and feedback mechanisms, operators can monitor the operational status of equipment remotely through an interactive interface without the need for on-site supervision. This approach enables precise online control, significantly reducing human risk in complex and hazardous environments. From an industrial demand perspective, the requirements for standardized and intelligent equipment operation in modern industry continue to rise. Traditional manual control modes are increasingly unable to meet the demands of high precision, high efficiency, and high safety in special operating conditions. Under the trend of digital transformation, optimizing equipment control processes through advanced human–machine interaction technologies to achieve efficient collaboration among humans, machines, and systems has become a key pathway for improving operational productivity. Therefore, the development and application of such a human–machine interaction system not only address the inherent limitations of traditional equipment control but also provide essential technical support for the digital and intelligent upgrading of complex equipment operations in special industrial scenarios. This demonstrates the system’s strong practical necessity and significant application potential.

To address the above pain points of live-line operation equipment control, the construction of a closed-loop digital twin operating system with precise command execution and real-time closed-loop feedback has become an inevitable choice, and this study is the first attempt to develop such a dedicated system for live-line operation equipment.

### 2.2 Overall architecture of the mixed reality-based closed-loop digital twin operating system for live-line operation equipment

**FIGURE 1:**
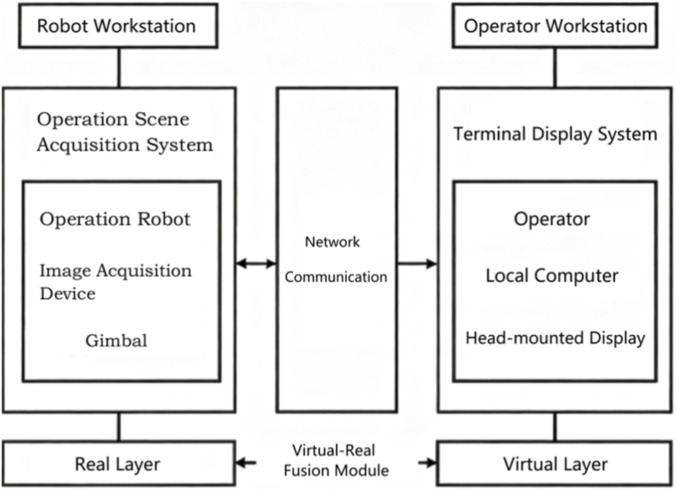
Framework of the MR-Based closed-loop digital twin operating system for live-line operation equipment.

By integrating digital twin and mixed reality technologies, we established a unified process framework for the first MR-based CLDTOS dedicated to live-line operation equipment, which enables the transformation of control intentions into executable device commands with full protocol compatibility for live-line tasks. Within the MR environment, the posture and position of the virtual robot model are continuously synchronized with those of the physical robot in the real world. Consequently, in actual operation scenarios, the operator only needs to wear an MR headset to visualize and monitor the robot’s movements through its virtual counterpart, even in cases where the real robot is difficult to observe directly. By interacting with the virtual model and using a handheld controller, the operator can manipulate and control the real device in real time, thereby enhancing the intuitiveness and precision of remote operation. As shown in Figure 1, the proposed system is divided into two main components: the active terminal and the passive terminal. The active terminal functions as the operator station, consisting of the operator, a head-mounted display (HMD), and a local computing unit. The local computer constructs a mixed reality (MR) environment that integrates stereoscopic visualization with an interactive human–machine interface. This MR scene provides the operator with a three-dimensional, immersive representation of the remote workspace and the corresponding interaction interface. By wearing the HMD, the operator can observe the robot’s posture in real time and execute control operations accurately within a safe and intuitive virtual environment, without direct physical exposure to the operational site. The passive terminal functions as the robotic operation station, consisting of a teleoperated robot, image acquisition devices, and a pan–tilt platform. Communication between the active and passive terminals is established through a self-organizing network device that integrates both video streaming and robot control protocols. This network framework enables the real-time transmission of visual data captured by onboard cameras back to the active terminal, providing the operator with auxiliary visual feedback to support situational awareness and decision-making during task execution (Lovon-Ramos, 2016).

**FIGURE 2:**
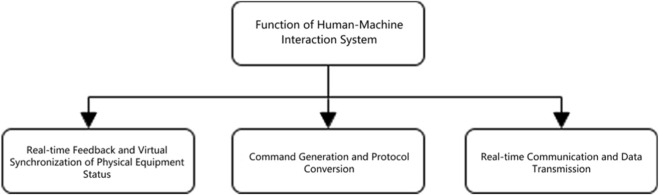
Function of human-machine interaction system.

As shown in Figure 2, the functions of the interactive system are mainly divided into the following three modules.

#### 2.2.1 Real-time feedback and virtual synchronization of physical equipment states

The real-time feedback and virtual synchronization module follows the core logic of “virtual command-driven, real-state feedback.” After the virtual control system issues operation commands to the physical equipment, multiple types of sensors deployed on the equipment and within the work environment continuously capture the actual operating conditions of the system. These include key information such as the progress of motion execution and variations in operational parameters. The real-state data collected are first subjected to preliminary processing to remove interference and standardize data formats before being transmitted to the virtual system. After receiving the processed data, the virtual system synchronizes the physical state of the real equipment to the virtual model according to predefined mapping rules, ensuring that the virtual representation accurately reflects the actual execution status of the physical device. Through the virtual interface, operators can directly observe the real-time response of the equipment to the issued virtual commands, promptly identify any deviations or anomalies, and use this information to adjust commands or optimize operations. In this way, a complete closed-loop workflow of “command issuance–physical execution–state feedback–virtual synchronization” is established.

#### 2.2.2 Command generation and protocol conversion

The command generation function serves as the critical link between virtual operations and the execution of real equipment. A comprehensive command template library covering various fundamental operation types is established within the system. When the operator performs an action in the virtual environment, the system automatically interprets the operator’s intention, extracts key operational parameters, and generates standardized virtual commands that conform to established task specifications and operational norms. Since real devices may operate under different control protocols and cannot directly interpret generalized virtual commands, the system incorporates a dedicated protocol conversion mechanism to translate standardized virtual commands into specific control signals recognizable by the corresponding physical equipment. During this conversion process, the system simultaneously performs compliance verification based on the hardware performance constraints of the target device. Commands that exceed operational limits or pose potential safety risks are automatically filtered out. When necessary, the system further optimizes or adjusts command parameters to ensure that the issued instructions not only meet task requirements but also guarantee the safe and stable operation of the real equipment.

#### 2.2.3 Real-time communication and data transmission

The real-time communication and data transmission module provides reliable support for both the delivery of virtual commands and the feedback of real equipment status data, serving as the foundation for ensuring system responsiveness and operational stability. The system adopts a hybrid communication architecture that integrates a data transmission bus with a dedicated wireless network. For short-range interactions between devices in the operational environment, the data bus enables high-speed data exchange, ensuring that control commands are rapidly transmitted to the device controller while real-time status information from the equipment is promptly fed back to the system. For long-distance data transmission scenarios, the system leverages the advantages of a dedicated wireless network to ensure timely and reliable data delivery, preventing desynchronization between virtual operations and real-world executions due to latency. In addition, to address potential interference or link failures in complex operational environments, this module incorporates a robust fault-tolerance mechanism. Data integrity is maintained through verification and redundancy protocols, ensuring uninterrupted transmission and preserving the continuity and stability of the entire operational process.

## 3 Development of a digital twin human–machine interaction system

### 3.1 Construction of the digital twin entity

The establishment of high-fidelity digital twin models for live-line operation robots and their working environment forms the core physical foundation of the MR-based closed-loop digital twin operating system for live-line operation equipment, which is the first time to build such a refined digital twin entity for the system of live-line operation equipment. The MR headset serves as the primary interface device. To achieve accurate model behavior, we hierarchically structured the DAE files of the robot into parent-child levels, and recalibrated and redefined the rotational axes. This configuration ensures that the robot model within the mixed reality environment can perform motion operations corresponding to its dual six-degree-of-freedom mechanical arms.

**FIGURE 3:**
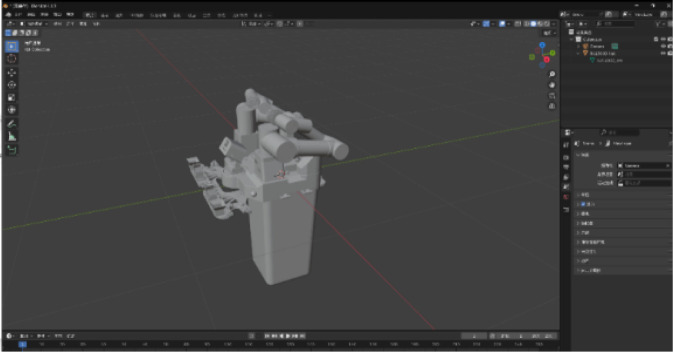
3D model optimization.

Based on the STL and DAE models, and in accordance with system requirements, the models were further optimized within the modeling software to enhance accuracy and dynamic behavior. In this study, Blender was selected as the primary tool for developing the three-dimensional equipment models. In the virtual environment, the motion of individual model components exhibits a certain degree of hierarchical dependency. To represent these dependencies, each model was divided into parent and child components, with the child components subordinated to their respective parent parts, thereby establishing a hierarchical motion structure within the virtual scene. For instance, the RJ1300D robotic model was decomposed into a base section, a left arm, and a right arm, with each of the two mechanical arms further subdivided into six degrees of freedom corresponding to their respective joint motions, as illustrated in Figure 3.

The Unity engine, as a real-time 3D content development platform, has been widely adopted across industries such as industrial manufacturing, education, and gaming (Qi, 2024). In this study, the system was developed based on the Unity engine, into which the virtual scene and interactive physical models were imported for further rendering and integration, enabling realistic visualization and dynamic interaction within the mixed-reality environment.

In the research on virtual model optimization and secondary rendering techniques, we established a full-process technical framework named “format parsing-parameter optimization-secondary rendering” to adapt to the display characteristics of MR devices. First, a dedicated DAE/STL format parsing module was developed to accurately extract the geometric topology, vertex coordinates, and basic attribute data of the original model. Meanwhile, redundant surfaces and vertices were processed to reduce the number of triangular meshes, thereby minimizing the rendering load on MR devices and improving real-time visualization performance. Subsequently, model parameter optimization for MR rendering was performed. Using color correction techniques, the color gamut of the models was adjusted—such as the reflective tones of metallic robotic arm components and the matte finishes of operation platforms—to align with the visual perception characteristics of MR environments. In combination with Physically Based Rendering (PBR) material design principles, specific material attributes were assigned to different model components, such as surface roughness for metallic parts and diffuse reflectance coefficients for non-metallic surfaces, thereby enhancing the visual realism and material fidelity of the virtual models. At the same time, a multi-level anti-aliasing technique, such as Fast Approximate Anti-Aliasing (FXAA), was introduced to eliminate edge aliasing and texture blurring in the models, thereby enhancing detail clarity and visual sharpness. As a result, high-precision models of the robotic arms, operation platforms, and other components adapted for the MR environment were generated. During the modeling process, point cloud data were simultaneously recorded to verify model accuracy, and marker data were stored for subsequent virtual–real alignment and calibration.

### 3.2 Mixed reality–based interactive environment simulation

**FIGURE 4:**
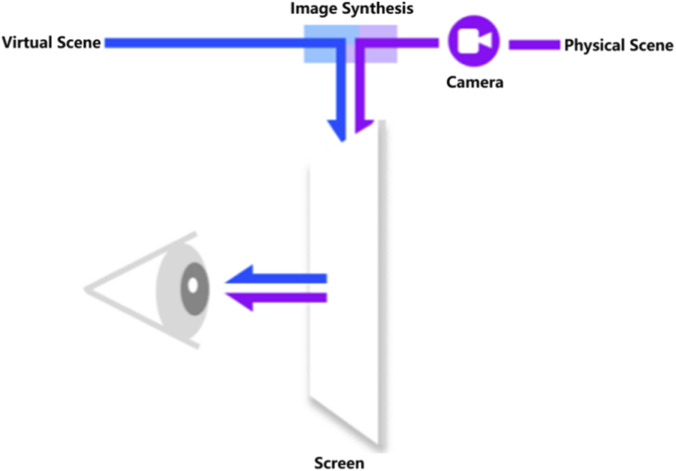
Video see-through scheme.

The construction of a high-fidelity and strongly interactive MR environment is an essential human-computer interaction layer of the closed-loop digital twin operating system for live-line operation equipment, and this study customizes the MR environment for the first time according to the operational characteristics of live-line equipment tasks. To meet the practical requirements of head-mounted display (HMD) operation in real work scenarios, the system establishes a dedicated MR environment in which only the necessary robotic models and user interface (UI) elements are rendered. This design ensures that the user’s field of view remains unobstructed during actual operation, as illustrated in Figure 4. In the MR environment, the pose of the virtual robot model remains continuously synchronized with that of the physical robot in the real world. This enables users to observe and verify the robotic arm’s position and orientation through the virtual model, even in situations where the actual robot is partially or fully obscured from view.

**FIGURE 5:**
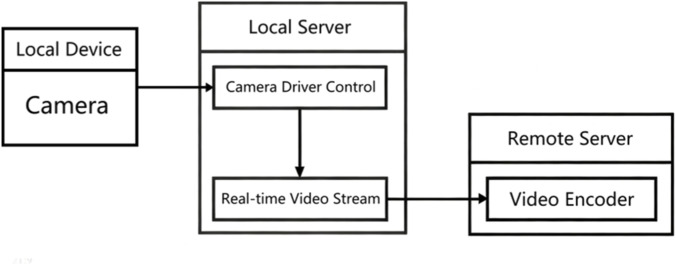
Image transmission scheme.

The user primarily perceives and interacts with the on-site task environment through camera-captured visual feedback. To enable efficient and low-latency communication, the system employs the gRPC video transmission protocol, with service interfaces and message types defined using Protocol Buffers (.proto) files. The Protoc compiler and corresponding gRPC plugins are used to generate both client and server-side interface code from these definitions, providing the structural framework for service invocation and request-response handling. The client obtains the server address via DNS resolution, establishes a TCP connection, and subsequently upgrades it to an HTTP/2 channel to support high-performance, bidirectional data transmission. Communication between the client and server is carried out over this established connection. The client reads the video stream, converts each frame into a byte stream, and encapsulates it into request messages according to the predefined message types. The server receives the transmitted video frame data through a request iterator, performing the corresponding decoding and processing operations in real time. The overall video transmission workflow is illustrated in Figure 5.

**FIGURE 6:**
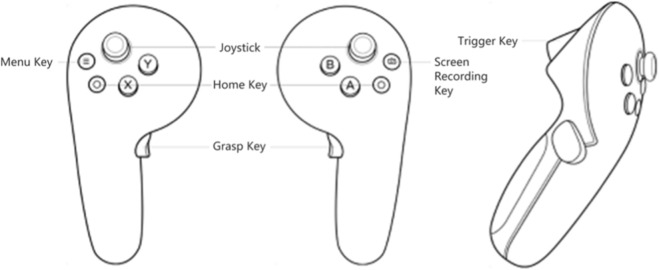
Controller button.

In this system, the robotic arm model is operated using dual PICO controllers, with button configurations illustrated in Figure 6.

During the interaction and user experience optimization phase, the focus was placed on enhancing both operational realism and immediacy of feedback. The MR controllers employ six-axis sensors to capture real-time hand motion angles and grip force, which are then precisely mapped to the joint rotations and tool actuation of the virtual robotic arm. When performing key operations—such as grasping wires or tightening cable clamps—the controllers generate vibration feedback of corresponding intensity. Combined with simulated wire bending and clamp engagement effects, this design effectively eliminates the “emptiness” often associated with virtual manipulation, thereby providing a more immersive and tactile interaction experience.

**FIGURE 7:**
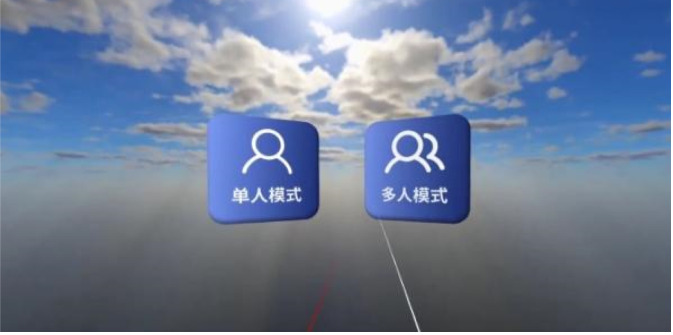
User mode selection interface.

According to the overall system architecture, a user interface was developed to support intuitive operation and mode management. Upon logging into the system, users can select between single-user mode and multi-user mode, as illustrated in Figure 7.

**FIGURE 8:**
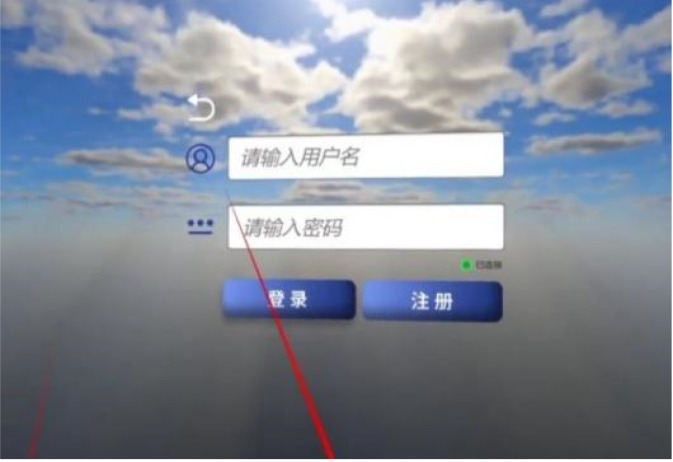
User login interface.

In the single-user mode, the user can directly proceed to task selection. In contrast, the multi-user mode requires user account authentication prior to access. If no account exists, the user must complete a registration process before continuing, as shown in Figure 8.

**FIGURE 9:**
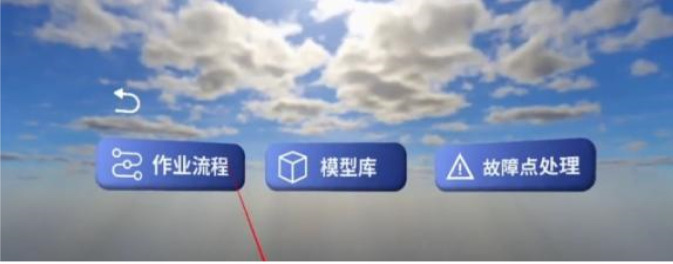
Task selection interface.

The task selection interface comprises three functional modules: Standard Operation Procedure (SOP), Model Library, and Fault Point Handling, as illustrated in Figure 9.

**FIGURE 10:**
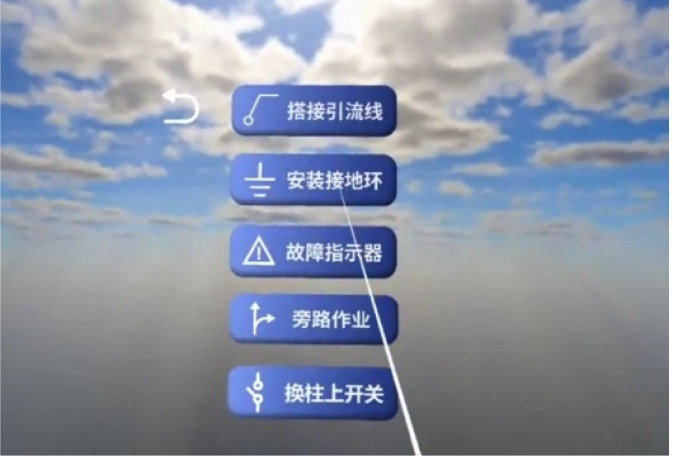
Standard operating procedure selection interface.

Among them, the Operation Procedure module provides five standard workflow options, including drainage wire connection, grounding ring installation, fault indicator maintenance, bypass operation, and pole-mounted switch replacement, as shown in Figure 10.

**FIGURE 11:**
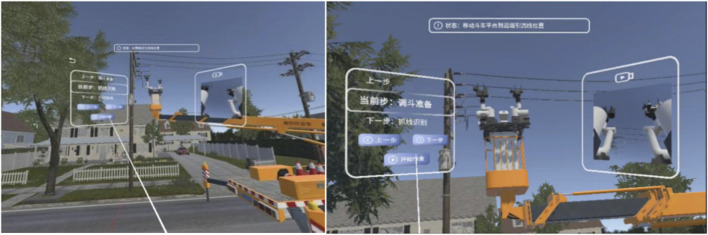
User interaction interface for wire connection operation.

After entering the Standard Operation Procedure task, the system interface displays information such as the current step, previous step, next step, task status, and a Start Operation button. Taking the lead wire connection task as an example, the interface layout is illustrated in Figure 11. During the lead wire connection task, under the unified scheduling and real-time closed-loop control of the closed-loop digital twin operating system, the bucket platform of the robotic arm is first driven to the target position with high precision. The left arm approaches the lead wire, passes through it with a wire stripping tool, and grasps and lifts the wire steadily. The right arm then adjusts its posture, passes through the lead wire, and completes stable grasping. After that, the left arm releases the lead wire and resets to provide sufficient operating space for the right arm. Subsequently, the bucket platform is adjusted to the distal main line position under the closed-loop feedback of the system. The left arm, equipped with the wire stripping tool, moves to the designated position and completes the wire stripping operation. Then, the right arm with the wiring tool carries the lead wire to the main line at the corresponding phase and performs the wiring operation. Under the closed-loop perception and precise control of the proposed digital twin operating system, the entire lead wire connection task is accomplished reliably and efficiently. This mode is used for engineering training and simulation.

**FIGURE 12:**
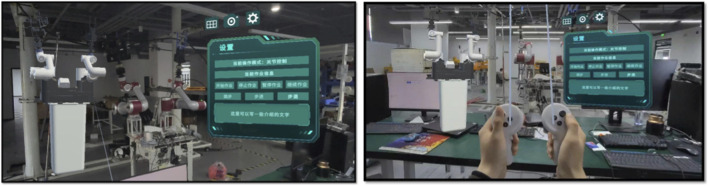
MR perspective mode.

The above content describes a demonstration and training mode based on a virtual scenario. However, after enabling the MR perspective mode, users can achieve two-way interaction with the real robotic arm—specifically, receiving information feedback from the real robot while independently controlling the robot to complete live-line operation tasks. In the perspective scenario, there is also a virtual robot model that responds to motion changes in real time. When it is difficult for users to observe the robot in the real world, they can confirm the pose of the robotic arm by observing the virtual robot model, as illustrated in Figure 12. The above MR perspective mode and lead wire connection process fully reflect the application of the closed-loop interaction system in live-line operation equipment tasks. As the first attempt to integrate such a closed-loop interaction system into live-line operation scenarios, it realizes real-time, controllable and correctable human-machine interaction, effectively solving the problems of poor observability and difficult adjustment in traditional live-line operations.

It is worth mentioning that this system supports simultaneous online interaction by multiple users, who can jointly observe and operate the live working process, thereby improving the efficiency and reliability of the operation procedure.

**FIGURE 13:**
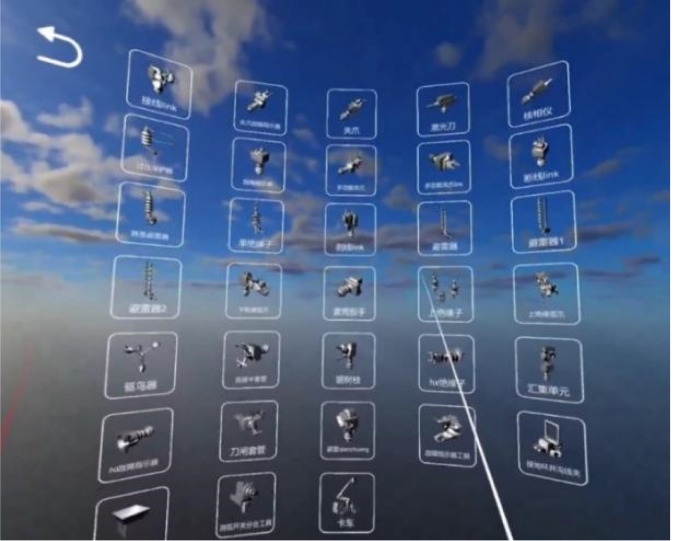
Model library module.

The system supports visualization of all tool models required in the operation workflow. More than thirty tool models are integrated into a unified model library, from which users can select and inspect specific tools as needed, as illustrated in Figure 13.

**FIGURE 14:**
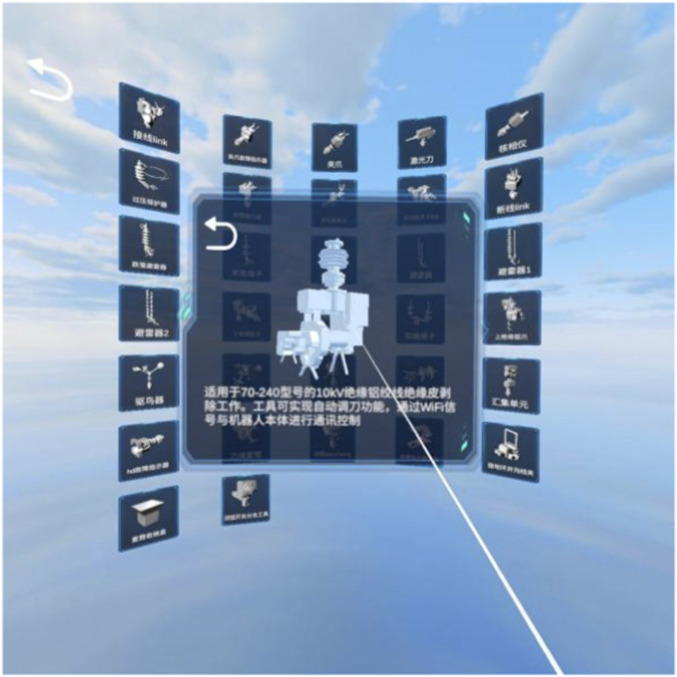
Model viewing.

After selecting a model, the system displays both the 3D model and its descriptive information. Users can manually rotate the model for detailed inspection and spatial understanding, as shown in Figure 14.

### 3.3 Human–machine interaction communication protocol

The real-time communication and data transmission module is the core data transmission layer of the closed-loop digital twin operating system for live-line operation equipment, providing a reliable underpinning for the delivery of virtual control instructions and feedback of physical device status for live-line tasks, which is the key to ensuring the real-time closed-loop operation of the system.

**FIGURE 15:**
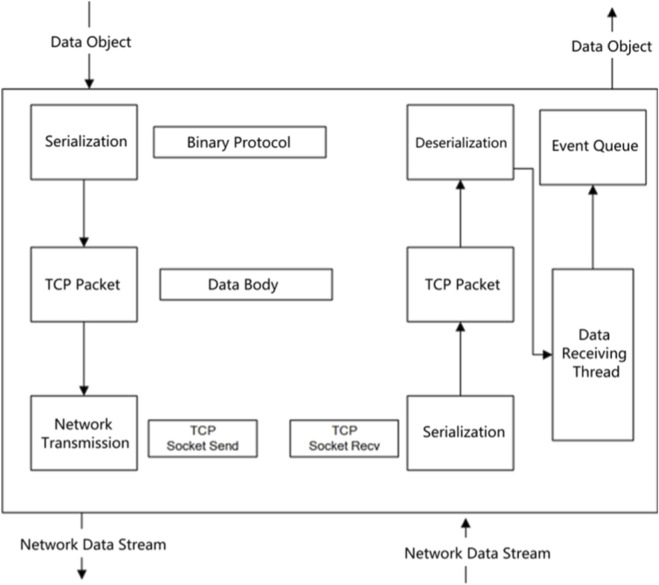
TCP protocol network architecture.

The system adopts a three-tier architecture consisting of a hardware control terminal, a communication server, and a physical device terminal. A bidirectional real-time channel based on the “instruction delivery-status feedback” mode is adopted for data transmission. Operational instructions are transmitted to the server via TCP data packets and then synchronized to the device for execution. The operational status of the device and the control terminal is transmitted back through the TCP reverse channel and displayed in real time, while the visual confirmation of operational actions is realized simultaneously (as shown in Figure 15).

**FIGURE 16:**
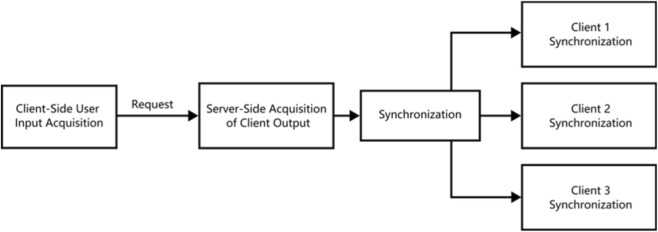
Inter-scenario message communication mechanism.

Communication between the server and clients is realized through the aforementioned TCP architecture. Key operations initiated by clients are executed by the server, and the execution results are synchronized to all associated clients to ensure system consistency (see Figure 16 for the inter-scenario message communication mechanism).

## 4 System usability analysis

In practical applications of the mixed reality (MR)–based digital twin human–machine interaction system, it is essential to evaluate the system’s usability across multiple dimensions.

**FIGURE 17:**
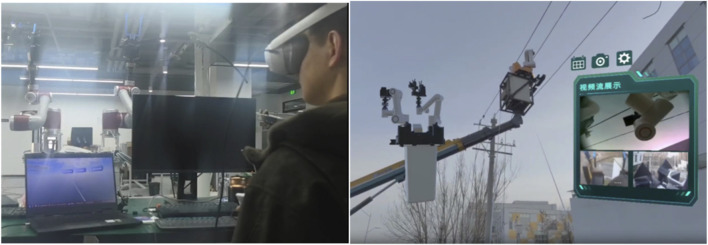
Outdoor testing of MR scenarios.

Since the human-machine interaction system is designed to issue real-time operational commands to physical robotic equipment in practical working environments, we need to evaluate the visibility and clarity of the MR scene under outdoor conditions, as illustrated in Figure 17. As shown in the figure, the virtual robot model displayed in the upper-left corner with a semi-transparent background frame allows clear observation of the specific posture of the robotic arm. Meanwhile, the real-world environment captured through the PICO passthrough camera video stream in the background remains clearly visible. As shown in the figure, the system can play an effective role in actual live-line working.

**FIGURE 18:**
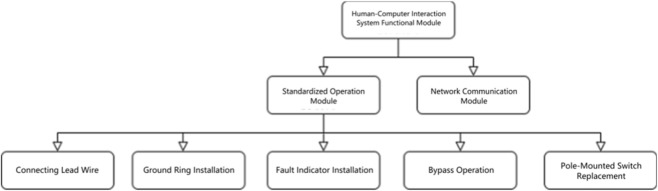
Human-computer interaction system functional module.

The mixed reality (MR)-based human–machine interaction system implements the standardized operation process module. This module focuses on the full-process standardization of five core live-line robotic operations—lead wire connection, grounding ring installation, fault indicator installation, bypass operation, and live replacement of pole-mounted switches. Guided by the design principles of “visualized progress, controllable operation, precise guidance, and real-time feedback”, the system deeply integrates command-interaction technology with standardized operation procedures. The system architecture consists of two main functional modules: the standardized operation module (as the primary module) and the network communication module, as illustrated in Figure 18.

**FIGURE 19:**
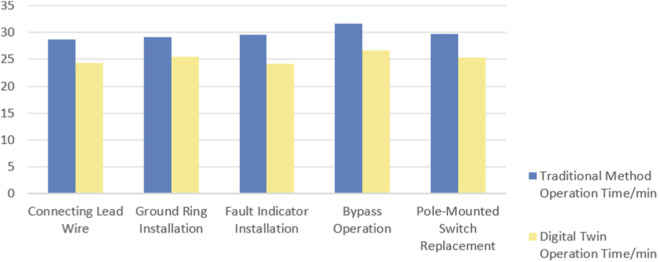
Human-computer interaction comparative experiment.

To further validate the improvement in operational efficiency achieved by the proposed digital twin-based human–robot collaborative operation system, a comparative experiment was conducted between the traditional control method and the digital twin-based operation approach. This experiment is the first to verify the application effect of the closed-loop digital twin operating system in five core live-line operation tasks of live-line equipment, and the subjects are required to operate the live-line operation equipment through the proposed system, which is different from the traditional open-loop control method. Both methods were tested under identical task conditions, following the standardized workflow of a live-line operation robot. The characteristics of the subjects were selected in accordance with those of frontline operators in real-world work scenarios: aged 20–30 years, with a predominance of male participants, who had no prior experience in industrial operations with MR devices (thus requiring training with the proposed system). The minimum sample size was calculated using G*Power software with an effect size of 0.5, α = 0.05 and 1-β = 0.8, yielding a value of 34. Considering factors such as equipment costs and personnel training expenses, 35 subjects were randomly selected from the population meeting the aforementioned criteria for the experiment. To mitigate the impact of the learning effect, the subjects were required to conduct sufficient task practice prior to the formal experiment. The task execution sequence for each subject was randomly assigned, and adequate rest intervals were provided between groups to eliminate the task sequence effect. After completing basic familiarization and training, participants performed interactive operations for the five standardized task procedures. The statistical results are presented in Table 1–3 and Figure 19. The t-values and p-values between different methods for each task were calculated via the significance test method. The p-values of all groups were less than 0.05, which indicates that the system proposed in this study exerts a statistically significant positive effect on the task completion efficiency of operators in special working conditions who require technical training. The efficiency improvement rates of each group were calculated, with a mean value of 14.3% and a 95% confidence interval of [12.5%, 16.1%], demonstrating a stable and significant improvement effect.

**TABLE 1:** Human-computer interaction comparative experiment (traditional method).

Traditional method	Average operation Time/min	Variance
Connecting Lead wire	28.9	0.68
Ground ring Installation	29.4	0.75
Fault indicator Installation	29.5	0.78
Bypass operation	31.2	0.82
Pole-mounted switch replacement	29.6	0.79

**TABLE 2:** Human-computer interaction comparative experiment (digital twin method).

Digital twin method	Average operation Time/min	Variance
Connecting Lead wire	24.2	0.59
Ground ring Installation	25.5	0.68
Fault indicator Installation	24.7	0.71
Bypass operation	26.5	0.75
Pole-mounted switch replacement	25.5	0.72

**TABLE 3:** Experimental data analysis.

Experimental tasks	t	p	Efficiency Improvement	Average Improvement	95% confidence interval for the mean
Connecting Lead wire	80.34	<0.05	16.3%	14.3%	[12.5%, 16.1%]
Ground ring Installation	70.02	<0.05	12.7%		
Fault indicator Installation	78.69	<0.05	13.3%		
Bypass operation	75.20	<0.05	15.1%		
Pole-mounted switch replacement	69.26	<0.05	13.9%		

## 5 Conclusion

In this study, to address the limitations of traditional open-loop control methods in operating live-line operation equipment—specifically poor collaborative efficiency—we for the first time propose a mixed reality MR-based closed-loop digital twin operating system and apply it to the task execution of live-line operation equipment. Building upon this framework, a human–robot collaborative digital twin system for live-line operation robots was further developed. The proposed system integrates MR-based interactive control, real-time data synchronization, and visualized operation guidance to enhance situational awareness and control reliability in complex working environments.

Comprehensive analysis and experimental validation of the interactive system were conducted. The experimental results demonstrated that, compared with traditional live-line robot control methods, the proposed system reduced task completion time by 14.3% on average, effectively enhancing efficiency in complex operational scenarios.

The human–robot collaborative digital twin system for live-line operation robots proposed in this study effectively addresses the challenges of poor collaborative efficiency commonly encountered in traditional control methods. By integrating mixed reality–based interaction and real-time digital twin synchronization, the system significantly enhances the efficiency of operator performance. This approach demonstrates substantial application potential for live-line operation robots and in the broader engineering implementation of robotic systems under special or complex working conditions.

## Data Availability

The original contributions presented in the study are included in the article/supplementary material, further inquiries can be directed to the corresponding author.
